# Much More Than the Malady: The Promise of a Web-Based Digital Platform Incorporating Self-Report for Research and Clinical Care in Mild Cognitive Impairment

**DOI:** 10.1016/j.mcpdig.2025.100224

**Published:** 2025-05-06

**Authors:** Andrew McGarry, Oliver Roesler, Jackson Liscombe, Michael Neumann, Hardik Kothare, Abhishek Hosamath, Lakshmi Arbatti, Anusha Badathala, Stephen Ruhmel, Bryan J. Hansen, Madeline Quall, Sandrine Istas, Arthur Wallace, David Suendermann-Oeft, Vikram Ramanarayanan, Ira Shoulson

**Affiliations:** a Department of Neurology, Cooper University Healthcare at Rowan University, Camden, NJ; b Modality.AI, Inc, San Francisco, CA; c San Francisco Veterans Affairs Health Care System, San Francisco, CA; d Janssen Research & Development, LLC, a Johnson & Johnson Company, New Brunswick, NJ; e ICON Strategic Solutions, ICON plc., Belgium; f Department of Anesthesia and Perioperative Care, University of California, San Francisco, CA

## Abstract

Traditional clinical trials in neurodegenerative disorders have utilized combinations of examination-based outcomes, global assessments by investigators and participants, and scales aimed at function, some of which are patient-reported outcomes. It is debatable whether these tools optimally convey therapeutic efficacy. A complementary approach using digital biomarkers to surpass exam-based limitations for detecting physical change coupled with a direct report from participants on what their sources of suffering are could be a useful advance in reporting beneficial effects of interventions, particularly if changes track together. We sought to determine the feasibility of remotely assessing speech, facial features, and cognition in an mild cognitive impairment (MCI) population, whether those extracted features could distinguish MCI from controls, and to explore what self-reported problems could reveal about the MCI experience. Our web-based platform was easy to use and revealed facial features in particular as capable of discriminating MCI from controls. Using the features that showed a statistically significant difference between cohorts (*P*<.01) produced an area under the receiver operating curve of 0.75. Self-reported problems with cognition, gait, sleep, and behavior were more common in the MCI group. The MCI was associated with 6 times more difficulty with falls (n=6 vs 1). These data support the feasibility and discriminative utility of using remote monitoring technology in combination with participant self-report in an MCI population. Future work will investigate the extent to which multimodal biomarkers combined with self-report can characterize MCI longitudinally and for potential research applications as a measure of therapeutic effect.

*Every patient you see is a lesson in much more than the malady from which he suffers.*

--William Osler, Aequanimitas “The Student Life” 1914:425.

At its core, medicine relies on the development of treatments whose efficacy benefits are reliably demonstrable and generalizable. Traditional clinical trials in neurodegenerative disorders have utilized combinations of examination-based outcomes, global assessments by investigators and participants, and scales aimed at function, some of which are patient-reported outcomes. Although there is standard experimental rigor in designing trials using these elements–an approach generally lauded as a strength of experimental therapeutics–whether or not the tools are optimal for determining what is effective is subject to debate. Examinations, for example, may reveal “how much” but not necessarily “so what”, and the domains in a functional scale, with ordinally ranked descriptions of disability, may only allow for partial description of participant experience. Adding to the dilemma, examination or structured scales may show neither “how much” nor “so what” in early populations of diseases characterized by prodromal phenotypic periods or slow progression, potentially making the study of critically important biological transitions in disease states difficult or impossible. Moreover, it does not necessarily follow that expert opinion as reflected in scale design will always capture what constitutes meaningful disability or improvement through the eyes of the participant—we might do well at characterizing the malady for assessment, but be less capable in attending to the illness experience in how we design the tools we use. Mindful of these challenges, a complementary approach using digital biomarkers to surpass exam-based limitations for detecting physical change coupled with a direct report from participants on what their sources of suffering are could be a useful advance in reporting the efficacy of interventions, particularly if changes track together.

Beyond the existing limitations in demonstration of efficacy, homogeneity of study populations and generalizability of results have been recurrent criticisms in clinical trial conduct, with National Institute of Health-led initiatives to improve access to trial participation.^1^^,^^2^ Among those at risk for access to clinical trials are veterans, a population with particular needs and challenges and for whom access to and trust in health care may be variable and complex.^3^^,^^4^ Awareness and attitudes toward research among veterans may also be complex; a recent survey showed only 58% knew what a clinical trial was, but trust in researchers was high and participation was of interest if their Veterans Affairs primary care physician recommended it.^5^ Because veterans represent a population for whom access to trials may be limited, we sought to test the feasibility of an easily accessible research platform in a veteran cohort with and without mild cognitive impairment (MCI). MCI describes cognitive decline that does not interfere with function or independence in comparison to people of similar age and educational background.^6^ Identifying people early in an MCI trajectory potentially allows for interventions before more substantial deterioration or clinical consequences. Therefore, there is a need for accurate, sensitive, noninvasive methods to improve the ascertainment of MCI, all the more crucial given the concerns for access to care and research in this population.

Several studies have reported the utility of speech and video signals for the assessment of MCI and other neurological conditions.^7^, ^8^, ^9^, ^10^, ^11^ Although these results are promising, it is not clear how generalizable they are due to several limitations. Accuracies reported for machine learning with small sample sizes are often overoptimistic.^12^^,^^13^ Many studies typically analyze a single modality (ie, text or speech) in isolation, as opposed to combining information from multiple modalities for greater discriminative power. Previous studies were either partly or wholly performed in-laboratory or in-clinic, with data collection technologies not built for larger-scale data acquisition. Although previous work has collected objective features to describe patient performance, digital capture of patient self-reporting on their current condition, capability, or well-being has not been done in unison with digital outcomes. This absence of asking patients to report directly on their status is consistent with traditional clinical trial methods, where investigators or participants must align their considerations and observations with the limited options in typical categorical scales. However, there is growing interest in prioritizing open-ended patient self-reports on what is bothersome and impairs function as the most meaningful way to follow disability progression and therapeutic response.^14^

Overall, an easily usable digital platform capturing metrics of interest and self-report that capably identify and describe a specific population would appear highly attractive for both efficacy evaluations and improved accessibility. We assessed a web-based multimodal platform that employed a virtual human guide to lead participants through several tasks while recording speech, text, facial, and cognitive measures, and unfiltered verbatim replies about bothersome problems and their functional consequences.^15^ To our knowledge, this is the first work in MCI that integrates both the measurement of objective biomarkers from multiple modalities and unfiltered patient verbatim replies about bothersome problems and their functional consequences into one comprehensive platform at scale. Our aims were to determine the feasibility of remotely assessing speech and cognition in MCI among veterans, whether extracted measures were informative at distinguishing MCI from controls, and what the most bothersome self-reported problems could reveal about the MCI experience.

## Participants and Methods

We recruited an age-and gender-matched comparative cohort of 100 MCI patients and 100 controls through the San Francisco Veterans Affairs Healthcare System. Key inclusion criteria were: (1) at least 55 years of age; (2) ability to provide informed consent; (3) possession of a valid phone number and email; (4) access to a smartphone, tablet, or personal computerwith internet connection and webcam; and (5) ability to read and speak in English. Key exclusions were: (1) formal diagnosis of dementia from any cause; (2) cognitive impairment due to cerebrovascular disease, head trauma, or alcohol/illicit substances; (3) diagnosis of Parkinson disease, schizophrenia, bipolar disease, or major depressive disorder; and (4) use of benzodiazepines, non-benzodiazepine receptor modulator sleeping medications, drugs for the treatment of Parkinson disease, or antipsychotics. The MCI participants were required to meet clinical diagnostic criteria of MCI (subjective concern for change; objective impairment in one or more cognitive domains; preservation of functional independence; no significant impairment in social or occupational engagement) and selected for screening based on appropriate ICD-10 coding for MCI in the electronic medical record.^16^

To determine whether measures extracted from digital data were clinically valid, we performed nonparametric Kruskal-Wallis tests for each metric to determine which showed statistically significant differences (α=0.01) between cohorts. To further investigate how well measures could discriminate between cohorts, we employed several classifiers for binary classification experiments using 5-fold cross-validation. We computed Pearson correlations between sessions to assess test-retest reliability and considered coefficients less than 0.5 unreliable. We used a Fisher’s Exact test to compare self-reported domains and complaints. Please see the Supplementary Material for methodological details.

The study was reviewed and approved by the institutional review board at the University of California, San Francisco. Prospective candidates were identified in the electronic medical record and e-mailed information about participation. If return correspondence was received, phone contact was then made for further explanation of study details and to answer any questions, followed by informed consent obtained electronically.

## Results

Recruitment was completed in 6 weeks. A virtual human (“Tina”) guided participants through 23 structured exercises in 2 sessions each one week apart to elicit speech, facial movements, and cognitive behaviors, including vowel phonations, counting on a single breath, read speech (sentences and Bamboo passage), spontaneous speech (picture description and open-ended questions), cognitive recall (immediate and delayed), digit span (forward and backward), a sequential commands task, and categorical fluency. The multimodal dialog platform automatically extracted 238 speech (eg speaking rate), 1450 facial (eg range and speed of movement of the lips), and 21 text (eg noun rate) features in near-real-time during all exercises of the interactive assessments (please see Supplementary Material for technical details of the platform assessment). We also asked participants to describe in their own words problems related to general health and personal well-being. They could indicate up to 5 problems for each category and could describe each problem for up to 3 minutes. Participants were asked to explain how problems affected daily functioning and what made them better or worse (when applicable). They completed a user experience survey at the end of each session (see Supplementary Material for Overview).

**Figure 1:**
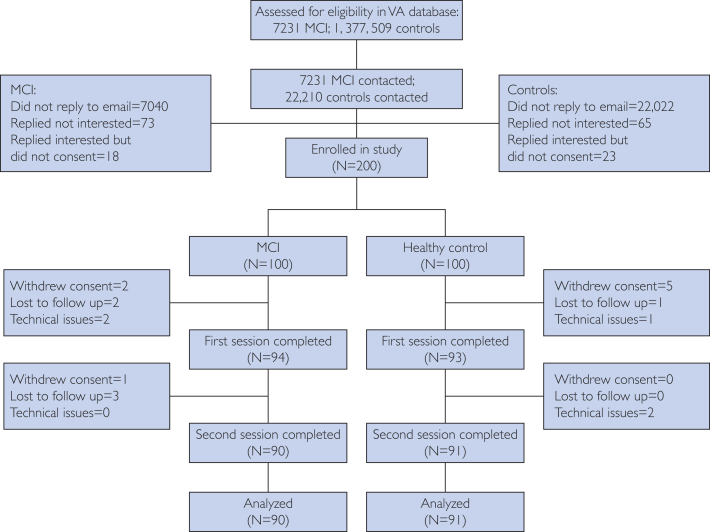
Disposition of participants.

**Figure 2:**
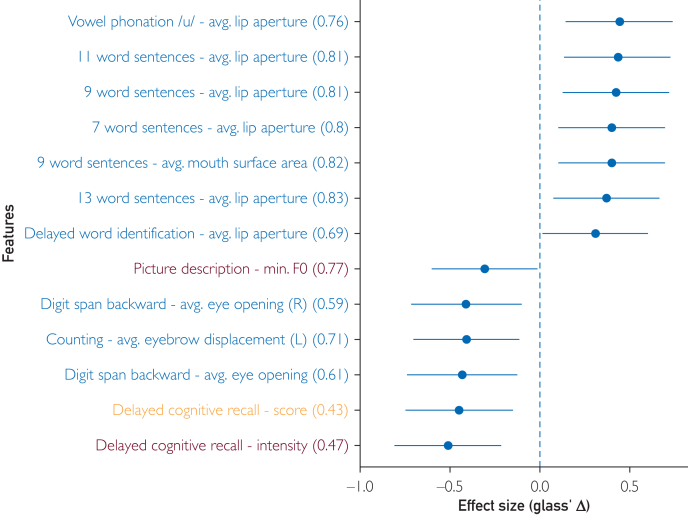
Effect sizes and test/retest reliabilities of speech, facial, and cognitive measures.

**Figure 3:**
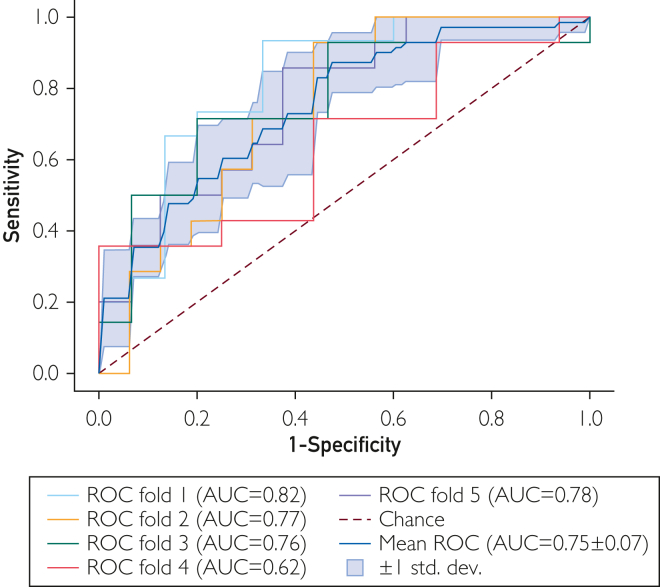
ROC curves for binary classification and 5-fold cross-validation using 13 measures with nominal difference between MCI and controls. ROC, receiver operating curve; MCI, mild cognitive impairment.

**Figure 4:**
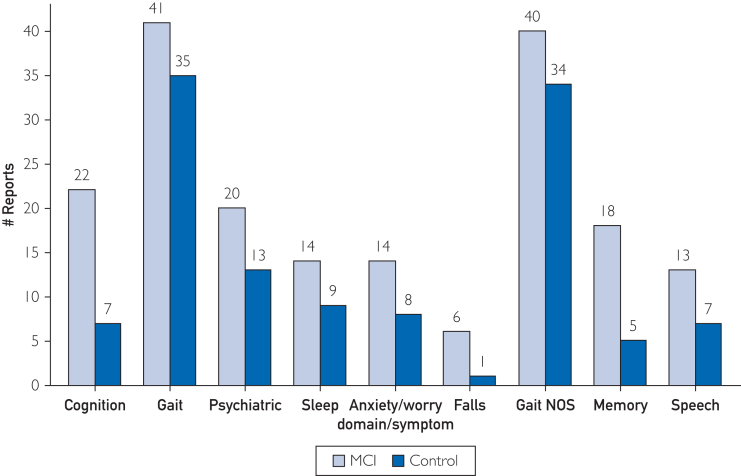
Domains and symptoms with a difference of more than 5 reports between groups.

**Figure 5:**
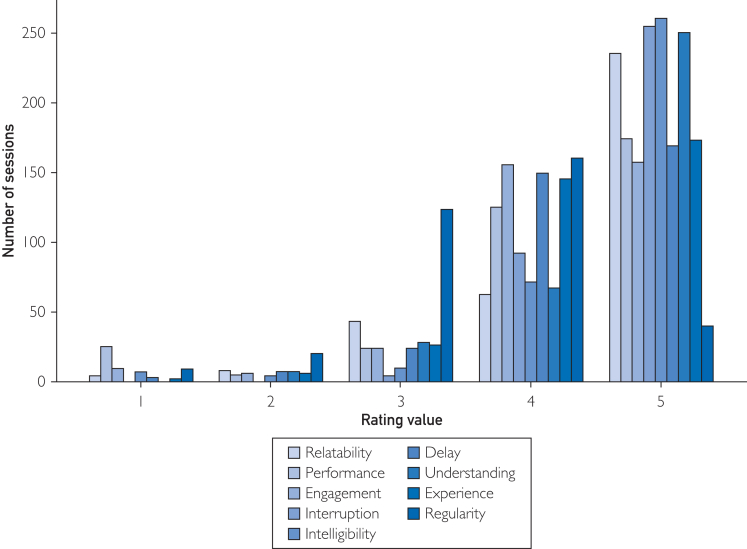
User survey results on Likert scale from 1 (very unsatisfactory) to 5 (very satisfactory).

Figure 1 shows participant disposition. For exploration, Figure 2 shows effect sizes and test-retest reliabilities for 13 significant differences between groups (*P*<.01, not corrected for multiplicity). Facial measures showed the strongest signal, accounting for 10 of the 13 measures (Figure 2). Measures related to mouth surface area and lip aperture were higher for MCI participants, whereas vertical eyebrow position and eye opening were lower. For picture description, the minimum F0 (fundamental frequency, a speech metric related to pitch) in MCI was lower compared to controls. Using measures with uncorrected significant difference between cohorts (*P*<.01) as input to a support vector machine classifier led to the best area under the receiver operating characteristics curve of 0.75 (Figure 3). Among self-reported domains, cognition was significantly different in the MCI group, as was the memory complaint among symptoms (22 vs 7, *P*=.008; 18 vs 5, *P*=.007). Figure 4 shows that MCI participants reported more problems with gait (n=41 vs 35), sleep (n=14 vs 9), and psychiatric items (n=20 vs 13). MCI was associated with 6 times more falls (n=6 vs 1) and more than twice as many problems with speech (n=13 vs 7). Figure 5 shows that most felt system performance, responsiveness, and overall experience were either satisfactory or very satisfactory (84.7%, 90.3%, and 90.3%).

## Discussion

Our data show the MCI veteran cohort effectively interacted with the platform and completed assessments. Recruitment was rapid, suggesting a remote digital platform may lend well to study participation. More than 89% felt engaged, liked their experience, and would use it again. Extracted biomarkers revealed that patterns in speech, countenance, and cognition may differentiate MCI from controls. It is notable that facial features, especially lip aperture, were reliable for discriminating MCI from healthy participants. This association between facial features and cognition in MCI may reflect an underappreciated breadth of the MCI neurodegenerative process. Persons for whom this distinction was possible were defined in the study as already having clinical MCI or not; whether the platform would remain sensitive for very early change, perhaps in the context of routine screening in older adults, is not yet known but an intriguing possibility. Further study may explore the platform’s utility for discriminating other neurodegenerative diseases from controls and perhaps demonstrating change in the context of therapeutic interventions.

Self-report data suggest that gait, sleep, and psychiatric symptoms may distinguish MCI with more diversity than commonly considered in clinic. Anxiety was more common in the MCI group, implying that functional difficulty and related concern may be more pervasive than the formal diagnostic criteria would suggest; indeed, MCI is a spectrum, and some participants would be expected to be evolving toward overt dementia. Consistent with this hypothesis, and perhaps expected, memory difficulty was substantially more common and significantly different in the MCI group. It is not trivial to consider that many people with MCI appear to be aware of their cognitive deficits, suggesting active surveillance for decline and potential intervention–not passive reaction to problems when they arise–should be clinical priorities. The MCI participants’ reports of speech as problematic are consistent with the literature, where language and speech patterns are known to change.^17^, ^18^, ^19^ The higher frequency of gait complaints is consistent with reports that numerous gait metrics in MCI can change (speed, cadence, variability, etc.), which may in turn worsen existing causes of gait dysfunction common in older adults.^20^, ^21^, ^22^, ^23^ It may be that the neurodegenerative process present in MCI, which garners attention for cognitive risk in the Alzheimer's Disease spectrum, influences other complex neurological domains like gait and is better considered as diffuse deterioration. Remarkably, falls were 6 times more common in the MCI group. Although falls are known in the literature to be more prevalent in MCI,^24^, ^25^, ^26^ current guidelines for MCI management do not include fall avoidance measures.^27^ Greater clinical awareness and surveillance for falling in MCI are warranted, and should be a central theme in future studies. Although exploratory, we found it striking that the self-report component of the platform was able to richly differentiate MCI participants—using their own words, in a setting of their choosing—where conventional clinical care, a sometimes harried and incomplete enterprise, may fail to do so.

This technology holds promise for applications in both research and clinical care. Future work in larger cohorts will investigate how well multimodal biomarkers combined with self-report can be used to differentiate MCI, characterize MCI severity and trajectory, perform in more diverse populations, and use additional feature sets/selection methods. The easy accessibility of the platform is especially intriguing for improving access in populations for whom traditional access to research is limited or absent and for whom traditional assumptions about what is important regarding disability and illness may be underreported or not altogether accurate.

## Conclusion

Ultimately, the enterprise of medicine must incorporate careful measurements of disease biology and phenotype as well as attention to the authentic, lived experience of our patients. Although modern medicine brings patients into clinics, laboratories, and hospitals for sophisticated assessments, it is notable that this distinction is by no means granted to all, nor does it follow that such assessments are necessarily timely, actionable, or complete. Much of human history, well before our centralized facilities, tools, and tests, has involved clinicians and healers going directly to the patient. Using a combination of technology capable of detecting the earliest phenotypic changes and direct inquiry into concerns of the patient in their natural environment, the digital platform tested here represents both the best of modern clinical surveillance capability and the tradition of meeting the patient as they are, where they are. Such capability holds promise to surpass current limitations in disease detection and understanding of the illness experience–indeed, much more than the malady.

## Potential Competing Interest

Drs Roesler, Liscombe, Neumann, Kothare, Hosamath, Arbatti, David Suendermann-Oeft, Shoulson, and Ramanarayanan are salaried and receive equity from Modality.AI, Inc. Dr Ruhmel, Hansen, and Quall are employees of Johnson and Johnson Innovative Medicine, New Brunswick, NJ, USA. Dr McGarry reports no competing interests.

## Ethics Statement

The study was reviewed and approved by the Institutional Review Board at the University of California, San Francisco. Informed consent was obtained for all participants.
