# Supplementary material for: Much More Than the Malady: The Promise of a Web-Based Digital Platform Incorporating Self-Report for Research and Clinical Care in Mild Cognitive Impairment
Source: Mayo Clin Proc Digit Health. 2025 May 6;3(2):100224. doi: 10.1016/j.mcpdig.2025.100224 (PMC12152880; doi:10.1016/j.mcpdig.2025.100224)
Supplement: Supplementary Material [file mmc1.docx]

**Additional Supplementary Material**

**eTable 1. Overview of Feasibility Survey**

| **Aspect** | **Question** | **Ratings** |
| --- | --- | --- |
| Performance | How would you rate the system’s overall performance? | 1: Very unsatisfactory, 2: Unsatisfactory, 3: Neutral, 4: Satisfactory, 5: Very satisfactory |
| Engagement | How engaged did you feel during the interaction? | 1: Highly disengage, 2: Disengaged, 3: Neutral, 4: Engaged, 5: Highly engaged |
| Delay | How satisfactory was the delay in Tina’s response to you? | 1: Very unsatisfactory, 2: Unsatisfactory, 3: Neutral, 4: Satisfactory, 5: Very satisfactory |
| Relatability | How relatable is Tina’s voice? | 1: Unrelatable, 2: Somewhat unrelatable, 3: Neutral, 4: Somewhat relatable, 5: Relatable |
| Understandability | How well do you think Tina understood you? | 1: Very badly, 2: Somewhat badly, 3: Neutral, 4: Somewhat well, 5: Very well |
| Regularity | How regularly would you use an app that involves interacting with Tina to monitor your health? | 1: Not at all, 2: Rarely, 3: Sometimes, 4: Often, 5: All the time |
| Experience | How would you rate your overall experience interacting with Tina? | 1: Very unsatisfactory, 2: Unsatisfactory, 3: Neutral, 4: Satisfactory, 5: Very satisfactory |
| Intelligibility | How intelligible was Tina? | 1: Very unclear, 2: Unclear, 3: Neutral, 4: Clear, 5: Very clear |
| Interruption | How often did Tina interrupt you? | 1: All the time, 2: Often, 3: Sometimes, 4: Rarely, 5: Not at all |

**eTable 2. Overview of Digitally Extracted Features**

| **Domain** | | **Features** |
| --- | --- | --- |
| Speech | Energy | shimmer (%), intensity (dB), signal-to-noise ratio (dB) |
|  | Timing | speaking and articulation duration (sec.), articulation  and speaking rate (WPM), percent pause time (PPT, %), canonical timing agreement (CTA, %) |
|  | Voice quality | cepstral peak prominence (CPP, dB), harmonics-to-  noise ratio (HNR, dB) |
|  | Frequency | mean, max., min. fundamental frequency F0 (Hz), first  three formants F1, F2, F3 (Hz), slope of 2nd formant  (Hz/sec.), jitter (%) |
| Facial | Mouth measurements | lip aperture/opening, lip width, mouth surface area, mean symmetry ratio between left and right half of the mouth |
|  | Movement | velocity, acceleration, jerk, and speed of lower lip and  jaw center |
|  | Eyes | number of eye blinks per sec., eye opening, vertical displacement of eyebrows |
| Text | Lexico-semantic | word count, percentage of content words, noun rate,  verb rate, pronoun rate, noun-to-verb ratio, noun-to-  pronoun ratio, closed class word ratio, idea density |
|  | Self-reported problems | reported symptoms, reported problem domains |
| Cognitive | Scores | percentage of correct words (immediate and delayed  word recall), digit span forward/backward score (ranges from 0 to 2) |
|  | Timing | response latency (sec.), response duration (sec.) |

**eTable 3. Glossary of extracted features. Features that showed nominally significant differences between cohorts (Figure 3a) are shown in bold**

| **Feature** | **Description** |
| --- | --- |
| shimmer | Shimmer (%) refers to the amplitude variation of the sound wave produced by the vocal cords from cycle to cycle. |
| **intensity** | Intensity (dB) is computed as the average intensity of the entire utterance in decibels. |
| signal-to-noise ratio | Signal-to-noise ratio (dB) is a measure of the strength of the desired signal relative to background noise (undesired signal). |
| speaking duration | Speaking duration (seconds) is the total duration (in seconds) of the utterance. |
| articulation duration | Articulation duration (seconds) is the duration (in seconds) of the utterance, removing all instances of stuttering-like disfluencies, other disfluencies, and pauses. |
| articulation rate | Articulation rate (words/minute) is the number of perceptually fluent words in each utterance divided by the duration (in minutes) of the utterance, removing all instances of stuttering-like disfluencies, other disfluencies, and pauses. |
| speaking rate | Speaking rate (words/minute) is the number of perceptually fluent words in each utterance divided by the total duration (in minutes) of the utterance, including pauses. |
| percent pause time (PPT) | Percent pause time (%) is the proportion of the total duration of all pauses to the total duration of the entire utterance. |
| canonical timing agreement (CTA) | Canonical Word Timing Alignment (%) is the percentage match in time alignments between an automated phonetic alignment of the utterance produced by the Montreal Forced Aligner (34) and Tina's canonical production of the same utterance. |
| cepstral peak prominence (CPP) | Cepstral peak prominence (dB) is an acoustic measure of voice quality and dysphonia severity. |
| harmonics-to-  noise ratio (HNR) | Harmonic-to-Noise Ratio (dB) is an assessment of the ratio between periodic components and non periodic components comprising a segment of voiced speech. |
| mean, max., **min. fundamental frequency (F0)** | Mean, maximum, and **minimum fundamental frequency (Hertz) over the entire utterance**. |
| first, second, third formants (F1, F2, F3) | The first, second, and third formants (Hertz) are the frequencies at which the first/lowest, second, and third spectral peak (or local maximum) occurs in the speech spectrum (resulting from an acoustic resonance of the human vocal tract). This is typically used to characterize vowel quality. |
| slope of 2nd formant | F2 Slope (Hz/sec) is the slope of the second formant F2 over the voiced utterance (typically a diphthong vowel) that correlates with dysarthria severity. |
| jitter | Jitter (%) refers to the frequency variation of the sound wave produced by the vocal cords from cycle to cycle. |
| **avg.**, min., and max. **lip aperture/opening** | **Average**, minimum, and maximum **lip aperture/opening, calculated as the Euclidean distance between the upper lip and lower lip (center).** |
| lip width | Average, minimum, and maximum lip width, calculated as the average Euclidean distance between right and left corners of the mouth. |
| **avg.** and max. **mouth surface area** | **Average** and maximum **total mouth surface area, calculated as the average** and maximum **area of the mouth opening**, respectively. |
| mean symmetry ratio between left and right half of the mouth | The mean symmetry ratio of the left and right sides of the mouth, measured as the ratio of the left to the right surface area of the mouth. |
| avg., min., and max. velocity, acceleration, jerk, and speed of lower lip and  jaw center | Average, minimum, and maximum velocity, acceleration, jerk, and speed of the lower lip and jaw center. |
| number of eye blinks per sec. (B, L, R) | The average number of eye blinks in blinks per second for the left and/or right eye. |
| **avg.** and max. **eye opening** (B, L, **R**) | **Average** and maximum **eye opening is calculated as the average of Euclidean distances between upper and lower extremities of** both eyes (B), the left eye (L), and **the right eye (R)**. |
| **avg.** and max. **vertical displacement of eyebrows** (B, **L**, R) | **Average** and maximum **vertical eyebrow displacement is calculated as the vertical distance between the positions of the left** and/or right **eyebrow center and the center of the inter-caruncular distance**. |
| word count | Total number of words in the utterance. |
| percentage of content words | Percentage of content words in the utterance. |
| noun rate | Number of nouns (incl. pronouns) divided by the total number of words in the utterance. |
| verb rate | Number of verbs divided by the total number of words in the utterance. |
| pronoun rate | Number of pronouns divided by the total number of words in the utterance. |
| noun-to-verb ratio | Total number of verbs divided by the total number of nouns. |
| noun-to-  pronoun ratio | Total number of pronouns divided by the total number of nouns. |
| closed class word ratio | Total number of closed class words (determiners, pronouns, conjunctions, prepositions, etc.) divided by open class words (nouns, verbs, adjectives, adverbs). |
| idea density | Sum of verbs, adjectives, adverbs, prepositions, and conjunctions divided by the total number of words. |
| reported symptoms | Number of times a specific symptom related to general health or personal well-being was reported. |
| reported problem domains | Number of times a specific problem domain related to general health or personal well-being was reported. |
| **percentage of correct words** (immediate and **delayed**  **word recall**) | Percentage of correctly recalled words (ignoring order). |
| digit span forward/backward score (ranges from 0 to 2) | A score of 2 was given if all digits were repeated in the correct order, a score of 1 if all digits were repeated but in a different order, and a score of 0 otherwise. |
| response latency (sec.) | Time (seconds) until the participant started talking after Tina finished speaking. |
| response duration (sec.) | Duration (seconds) of participant’s response. |
